# Deletion Mutants of the Attenuated Recombinant ASF Virus, BA71ΔCD2, Show Decreased Vaccine Efficacy

**DOI:** 10.3390/v13091678

**Published:** 2021-08-25

**Authors:** Elisabeth Lopez, Laia Bosch-Camós, Elizabeth Ramirez-Medina, Elizabeth Vuono, Maria Jesus Navas, Marta Muñoz, Francesc Accensi, Jinya Zhang, Uxia Alonso, Jordi Argilaguet, Maria Luisa Salas, Nikolay Anachkov, Douglas P. Gladue, Manuel V. Borca, Sonia Pina-Pedrero, Fernando Rodriguez

**Affiliations:** 1 IRTA, Centre de Recerca en Sanitat Animal (IRTA-CReSA), Campus de la Universitat Autonoma de Barcelona, 08193 Bellaterra, Spain; elisabeth.lopezf@gmail.com (E.L.); Laia.Bosch@irta.cat (L.B.-C.); mariajesus.navas@irta.cat (M.J.N.); Marta.munoz@irta.cat (M.M.); Francesc.accensi@irta.cat (F.A.); jinya.zhang@irta.cat (J.Z.); Uxia.alonso@irta.cat (U.A.); jordi.argilaguet@irta.cat (J.A.); 2 USDA Plum Island Animal Disease Center, Agricultural Research Service, United States Department of Agriculture, Greenport, NY 11944, USA; Elizabeth.Ramirez@usda.gov (E.R.-M.); Elizabeth.Vuono@usda.gov (E.V.); Douglas.Gladue@usda.gov (D.P.G.); Manuel.Borca@usda.gov (M.V.B.); 3 Departament de Sanitat i d’Anatomia Animals, Facultat de Veterinària, UAB, 08193 Bellaterra, Spain; 4 Centro de Biología Molecular Severo Ochoa, Consejo Superior de Investigaciones Científicas, Campus de la Universidad Autònoma de Madrid, 28049 Madrid, Spain; mlsalas@cbm.csic.es; 5 Biologics Development, Huvepharma, 3A Nikolay Haytov Street, 1113 Sofia, Bulgaria; n_anachkov@biovet.com

**Keywords:** African swine fever (ASF), African swine fever virus (ASFV), live attenuated virus (LAV), vaccine, virulence factor, double mutant, protection

## Abstract

African swine fever (ASF) has become the major threat to the global swine industry. Lack of available commercial vaccines complicates the implementation of global control strategies. So far, only live attenuated ASF viruses (ASFV) have demonstrated solid protection efficacy at the experimental level. The implementation of molecular techniques has allowed the generation of a collection of deletion mutants lacking ASFV-specific virulence factors, some of them with promising potential as vaccine candidates against the pandemic genotype II ASFV strain currently circulating in Africa, Europe, Asia and Oceania. Despite promising results, there is room for improvement, mainly from the biosafety point of view. Aiming to improve the safety of BA71∆CD2, a cross-protective recombinant live attenuated virus (LAV) lacking the ASFV CD2v gene (encoding β-glucuronidase as a reporter gene) available in our laboratory, three new recombinants were generated using BA71∆CD2 as a template: the single mutant BA71∆CD2*f*, this time containing the fluorescent mCherry reporter gene instead of CD2v, and two double recombinants lacking CD2v and either the lectin gene (EP153R) or the uridine kinase (UK) gene (DP96R). Comparative in vivo experiments using BA71∆CD2*f*, BA71∆CD2DP96R and BA71∆CD2EP153R recombinant viruses as immunogens, demonstrated that deletion of either DP96R or EP153R from BA71∆CD2*f* decreases vaccine efficacy and does not improve safety. Our results additionally confirm ASFV challenge as the only available method today to evaluate the protective efficacy of any experimental vaccine. We believe that understanding the fine equilibrium between attenuation and inducing protection in vivo deserves further study and might contribute to more rational vaccine designs in the future.

## 1. Introduction

Lack of commercially available vaccines complicates the control of African swine fever (ASF), today’s number one threat to the global swine industry [1]. The complexity of African swine fever virus (ASFV), the causative agent of ASF, together with the limited resources historically invested in this disease, has delayed vaccine development. This situation has dramatically changed with the last re-entrance of the virus in Europe in 2007 and the subsequent spread to the West, reaching the European Union in 2014, and to the East, reaching China in 2018. It has been shown that classically inactivated vaccines for ASF are ineffective, independent of the adjuvant tested [2]. To date, all subunit vaccines that have been reported have failed at inducing solid protection against the pandemic ASFV (Georgia2007/1), despite being the ideal choice in the long term if one becomes available [3]. So far, only ASF live attenuated viruses (LAVs) have conferred solid protection against experimental challenge with homologous viruses, with both antibodies and T cells, playing important roles in the protection afforded [4,5]. These studies have been performed with either natural attenuated ASFV isolates or with recombinant LAVs obtained by specific deletions of virulence-associated genes from the homologous virulent virus [6,7,8,9,10,11]. Additionally, our previous studies have demonstrated the cross-protective ability of BA71∆CD2, a deletion mutant lacking the CD2v (encoding β-glucuronidase as a reporter gene) from the genotype I virulent BA71 ASFV strain, to confer protection against both parental BA71 and genotype II Georgia2007/1 experimental challenges [12]. Despite its protective potential and the ability to grow in stable cell cultures (Cos-1 cells) without genetic changes, a small percentage of BA71∆CD2-immunized pigs showed low, but detectable, amounts of ASFV DNA in sera [12]. Aiming to reduce the residual viral load observed with BA71∆CD2 and increase the safety of our vaccine prototype, three new recombinant viruses were generated using the same methodology and substituting the ASFV ORFs with fluorescent markers: the single mutant BA71∆CD2*f*, this time containing the fluorescent mCherry marker instead of CD2v, and two double recombinants lacking CD2v and a second virulence-associated gene, either DP96R, encoding the ASFV uridine kinase gene [8], or EP153R, encoding the ASFV lectin [13]. Once generated, the two double mutants, BA71∆CD2DP96R and BA71∆CD2EP153R, were used in a comparative in vivo immunization study with their parental fluorescent counterpart, BA71∆CD2*f*, whereas the control group of the whole experiment was immunized with PBS. Finally, all pigs were challenged with the pandemic Georgia2007/1 strain. The specific antibody and T-cell responses induced, together with the presence of ASFV in pig sera before and after challenge, determined that the additional deletion of DP96R or EP153R from BA71∆CD2*f* did not improve the safety of the vaccine, but instead reduced the protective potential against Georgia2007/1 challenge. These studies confirm previous results obtained with other recombinant LAVs designed using genotype II and I ASFV strains as templates [14,15,16,17,18]. The lack of exact correlation between in vitro assays and protection in swine confirms ASFV challenge as the only valid method to accurately evaluate the protective efficacy of any experimental vaccine approach. Understanding the mechanisms governing the efficacy of LAVs might become essential to design safer vaccine approaches against ASF in the future.

## 2. Methods

### 2.1. Cells and Viruses

Porcine alveolar macrophages (PAMs) were obtained by successive bronchoalveolar lung lavages with PBS. Macrophage cell culture was performed with RPMI 1640 medium (Life Technologies, Grand Island, NY, USA), supplemented with 10% fetal bovine serum (HyClone, GE Healthcare, Chicago, IL, USA).

The recombinant viruses were purified by limited dilution on PAMs. The virulent isolate Georgia2007/1, used for challenge purposes, was kindly provided by Dr. Linda Dixon (The Pirbright Institute, Ash Road, Pirbright, Surrey GU24 0NF, UK) and titrated in porcine macrophages.

### 2.2. Generation of the Recombinant Deletion Mutants and Comparative In Vitro Growth Curves

**Figure 1:**
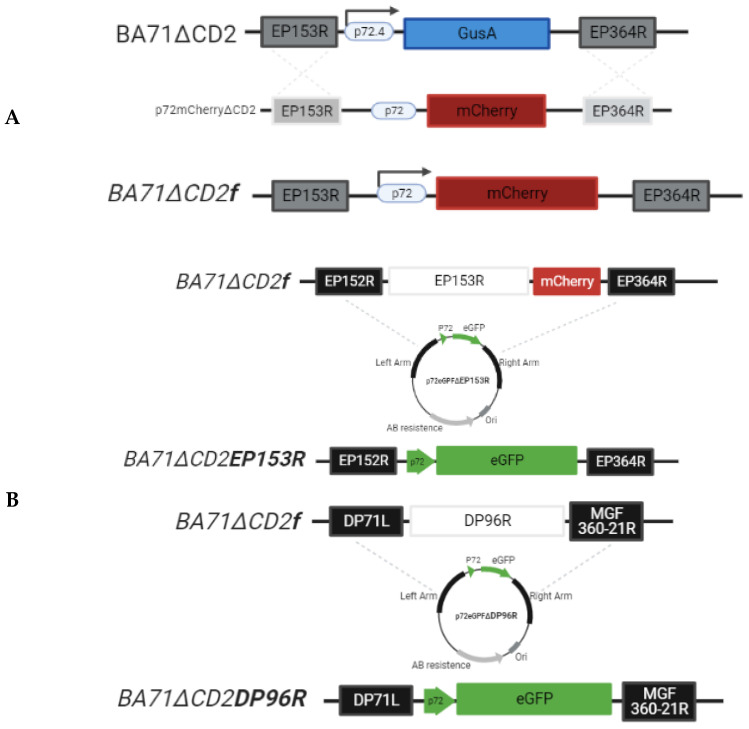
Schematic representation of the recombinant parental virus BA71∆CD2*f* (**A**) and the EP153R and DP96R gene regions deleted by homologous recombination in recombinant viruses BA71∆CD2EP153R and BA71∆CD2DP96R (**B**). Dotted lines indicate the homologous regions between the parental virus and the correspondent transfer plasmid.

For comparative purposes, we first generated a fluorescent BA71∆CD2*f* by homologous recombination (Figure 1A), following a similar procedure described before [19]. The right and left genome arms of the CD2 locus (approximately 700–800 bp in length) were obtained by DNA synthesis at Epoch Life Sciences (Sugar Land, TX, USA) to construct the p72mCherry∆CD2 transfer vector encoding the mCherry reporter gene under the ASFV p72 late promotor. Macrophage cell cultures were infected with the parental virus BA71∆CD2 (containing a β-glucuronidase, encoded by the GusA gene, as a reporter gene) and transfected with the p72mCherry∆CD2 recombination transfer vector. By homologous recombination, the GusA gene was exchanged by the mCherry reporter gene, generating the recombinant BA71∆CD2*f*.

Next, we generated the recombinants BA71∆CD2DP96R and BA71∆CD2EP153R, using BA71∆CD2*f* as the parental virus and p72eGFP∆DP96R or p72eGFP∆EP153R as transfer vectors, respectively (Figure 1B). Both transfer vectors harbor the green fluorescent protein (eGFP) gene under the control of the ASFV p72 late gene promoter as a reporter gene cassette, flanked by the left and right homologous arms of the deleted ORFs to allow homologous recombination, following the same system described above. 

The recombinant viruses were obtained after successive rounds of limit dilution purification on macrophage cell cultures, followed by a full genome sequencing to ensure the genome integrity and the designed deletions.

Comparative in vitro growth curves of BA71∆CD2*f*, BA71∆CD2DP96R and BA71∆CD2EP153R were performed by infecting PAMs at a multiplicity of infection (MOI) of 0.1. After 2 h of adsorption at 37 °C in 5% CO_2_, the inocula were removed and replaced with fresh media. The supernatants were harvested at 16, 24 and 48 h post infection (hpi). Virus loads in supernatants were quantified by qPCR following previously described methods [20] and the percentage of infected cells at each time point was followed by flow cytometry, detecting the eGFP or mCherry reporter genes.

### 2.3. Animals, Hosting and Welfare

Six-to-eight-week-old male (Large White × Landrace) pigs, ranging from 15 to 30 kg, were housed in the BSL-3 facilities of Institut de Recerca i Tecnologia Agroalimentària (IRTA)-CReSA (Bellaterra, Spain). Animal experiments were conducted according to animal welfare ethics and protocols approved by the Ethics Commission in Animal Experimentation of the Generalitat de Catalunya (code CEA-OH/9212/2, in accordance to the European (Directive 2010/63/EU) and Spanish (Real Decreto 53/2013) regulations under the supervision of the IRTA’s Ethical and Animal Welfare Committee. 

### 2.4. In Vivo Experimental Approach

Twenty-four pigs were divided into four groups of six pigs each, without any contact between the groups. After one week of acclimation, each pig was intramuscularly immunized once with 1 mL of either PBS (control) or 10^6^ plaque-forming units (PFU) of either BA71∆CD2*f*, BA71∆CD2EP153R or BA71∆CD2DP96R. 

Twenty-four days later, all pigs were intramuscularly challenged with 10^3^ gene equivalent copies (GEC) of the Georgia2007/1 ASFV virulent strain, equivalent to 10^3^ hemadsorbing units (HAU). Pigs were bled at days 0, 4, 7, 14 and 24 after immunization (dpi) and after Georgia2007/1 challenge (dpc) to follow both the kinetics of virus load by qPCR and the ASFV-specific antibody and T-cell responses. Rectal temperature and clinical signs were recorded daily, following guides previously published [21]. Post-mortem examinations were carried out to confirm or discard the presence of ASF-compatible pathological lesions.

### 2.5. Analytical Methods

Experimental immunization, clinical observations, immunological assays and virus titration methods were previously described [12,21] and performed as briefly described below. 

#### 2.5.1. Antibody Detection by ELISA

Antibodies in pig sera were quantified by the OIE-approved indirect ELISA, based on soluble ASFV-infected cell-extract-coated plates, generously provided by Dr. Carmina Gallardo (EU ASF reference laboratory, CISA-INIA, Madrid, Spain). The presence of positive sera was detected using a peroxidase-conjugated anti-pig immunoglobulin G (IgG) at 1:20,000 dilution (Sigma-Aldrich, St. Louis, MO, USA) as secondary antibody and soluble 3,3′,5,5′-tetramethylbenzidine (TMB) as specific peroxidase substrate (Sigma-Aldrich). Reactions were stopped with 1 N H_2_SO_4_ (Sigma-Aldrich), and the ELISA plates were read at an optical density of 450 nm (OD_450_). 

#### 2.5.2. T-Cell Response by ELISPOT Assay

The frequency of ASFV-specific interferon gamma-secreting cells (IFNγ-SC) in peripheral blood mononuclear cells (PBMCs) was analyzed by an enzyme-linked immunosorbent spot (ELISPOT) assay using commercial monoclonal antibody tandems (swine IFNγ; Cytoset). Briefly, PBMCs were isolated from whole blood by density-gradient centrifugation with Histopaque 1077 (Sigma-Aldrich). For PBMC cultures, RPMI 1640 medium supplemented with 10% fetal bovine serum (HyClone, GE HealthCare), 50,000 IU penicillin/L (*Invitro*gen), and 50 mg streptomycin/L (*Invitro*gen) was used. Trypan blue was used to assess cell viability. PBMCs were specifically stimulated for 20 h in vitro with different ASFV isolates at a MOI of 0.2. RPMI, and 10 µg/mL of phytohemaglutinin (PHA, Sigma-Aldrich) was used as the control of the technique. Any sample scoring ≥300 spots/500,000 PBMCs received a score of 300, which was considered the limit of our assay resolution. 

#### 2.5.3. Virus Quantification

Titrating ASFV by hemadsorption is not always possible when working with non-hemadsorbing strains, such as BA71∆CD2. Aiming to adopt a reliable comparative method for ASFV titration of multiple samples, pig sera, collected at different time points during the experiment, were used to quantify the ASFV virus DNA by real-time qPCR [20]. Due to the fact that our qPCR technique had previously shown more reproducible results in sera than in whole blood, virus in sera and no viremia was measured, being aware that the amount of virus present in blood after challenge with Georgia2007/1 would be around 1 log higher in magnitude. Briefly, the viral genomic DNA was obtained from 200 µL of sera using the Nucleospin Blood kit (Macherey-Nagel, Düren, Germany) and then used as template to amplify an 85 bp-long fragment from the ASFV serine protein kinase gene (R298L). PCR amplifications were performed in duplicate using the corresponding standards for absolute quantification. Results are expressed as log10 GEC per mL of sera. The detection limit of the technique was set at 10^3^ GEC/mL. 

## 3. Results and Discussion

### 3.1. Replication of Recombinant Viruses in Swine Macrophage Cultures

**Figure 2:**
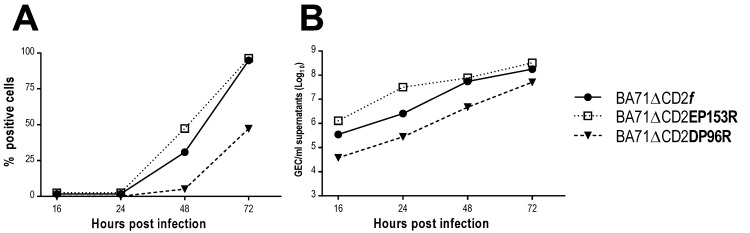
Comparative infection kinetics of the three recombinant viruses, BA71∆CD2*f*, BA71∆CD2EP153R and BA71∆CD2DP96R, in PAMs. (**A**) Percentage of infected cells analyzed by flow cytometry and (**B**) ASFV DNA amount found in the supernatant of infected PAMs at different time points after infection.

PAMs infected with either BA71∆CD2*f* and BA71∆CD2EP153R followed indistinguishable kinetics of infection (Figure 2). As expected, more than 95% of the PAMs were infected by 72 h (hpi) (Figure 2A), showing also similar maximum titers of ASFV-DNA in their supernatant (Figure 2B). Conversely, at the same multiplicity of infection (MOI), BA71∆CD2DP96R showed a delay in the infection kinetics, with only half of the PAMs becoming infected at 72 hpi and showing a maximum titer one log below of those achieved by BA71∆CD2*f* and BA71∆CD2EP153R. 

### 3.2. Comparative Protective Efficacy Induced by BA71∆CD2EP153R and BA71∆CD2DP96R Recombinant Viruses

**Figure 3:**
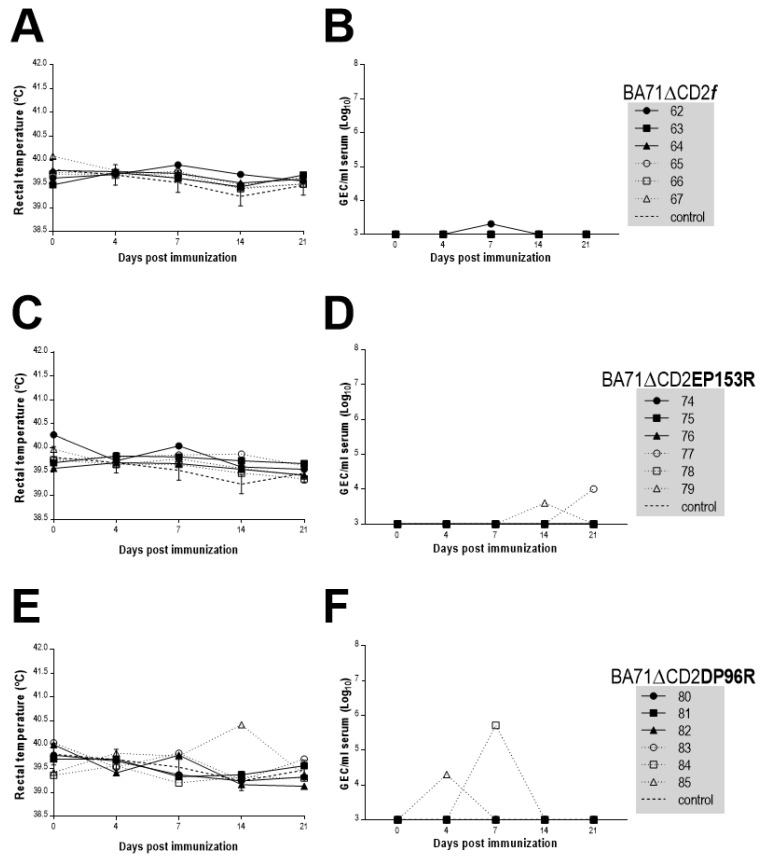
Rectal temperature and ASFV DNA amount found in sera after immunization with BA71∆CD2*f* (**A**,**B**) BA71∆CD2EP153R (**C**,**D**) or BA71∆CD2DP96R (**E**,**F**) in each individual animal. Average and standard deviation values from control pigs immunized with PBS are shown. ASFV DNA amounts are plotted on a logarithmic scale as GEC per milliliter of serum, 10^3^ GEC/mL being the limit of detection of the assay.

Aiming to confirm if the deletion of DP96R or EP153R reduced the residual viral load previously observed after immunization with BA71∆CD2 [12], the amount of ASFV DNA of each recombinant virus was followed directly after inoculation until the day of the Georgia2007/1 challenge (Figure 3). No clinical signs were observed following the immunization. The virus titers of BA71∆CD2*f*-immunized animals before challenge, as well as the protection afforded, yielded similar results to our previous studies [12], with the exception of pig number 66, euthanized at 6dpc (Figure 5), which responded poorly to the vaccine (Figures 7A and 8) and was Unfortunately, two out of the six pigs immunized with either BA71∆CD2EP153R or BA71∆CD2DP96R showed low albeit detectable copy numbers of virus genome in sera, confirming that these new prototypes do not improve the safety of BA71∆CD2. 

Twenty-four days after immunization, all pigs were intramuscularly challenged with a lethal dose of 10^3^ GEC of the highly pathogenic Georgia2007/1 isolate. 

**Figure 4:**
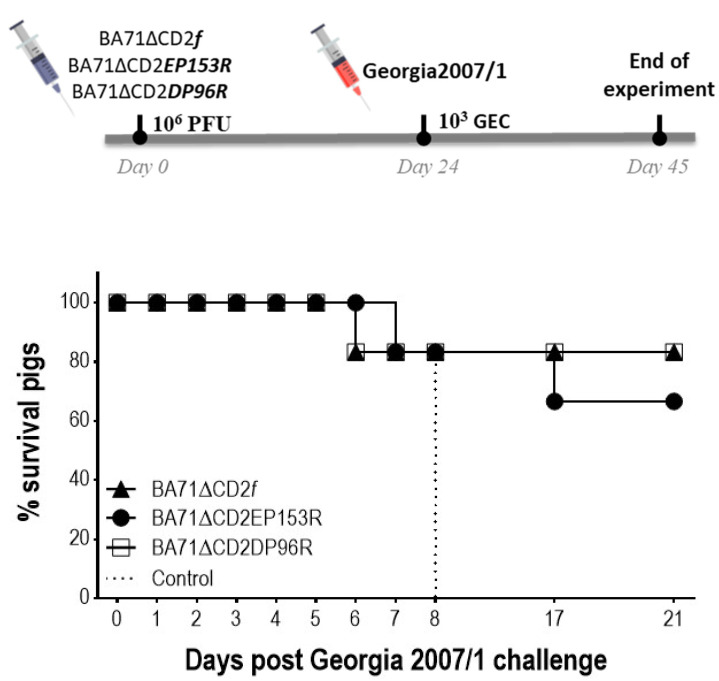
Survival rates after Georgia2007/1 challenge. Pigs were immunized with either BA71∆CD2*f*, BA71∆CD2DP96R or BA71∆CD2EP153R and then challenged 24 days later with a lethal dose of Georgia 2007/1.

Control pigs were euthanized by day 8 post-challenge, while only one out of the six pigs vaccinated with BA71∆CD2*f* (animal 66) died after Georgia2007/1 challenge (Figure 4). One and two out of the six pigs inoculated with BA71∆CD2DP96R and BA71∆CD2EP153R, respectively, did not survive the lethal ASFV challenge (Figure 4). 

**Figure 5:**
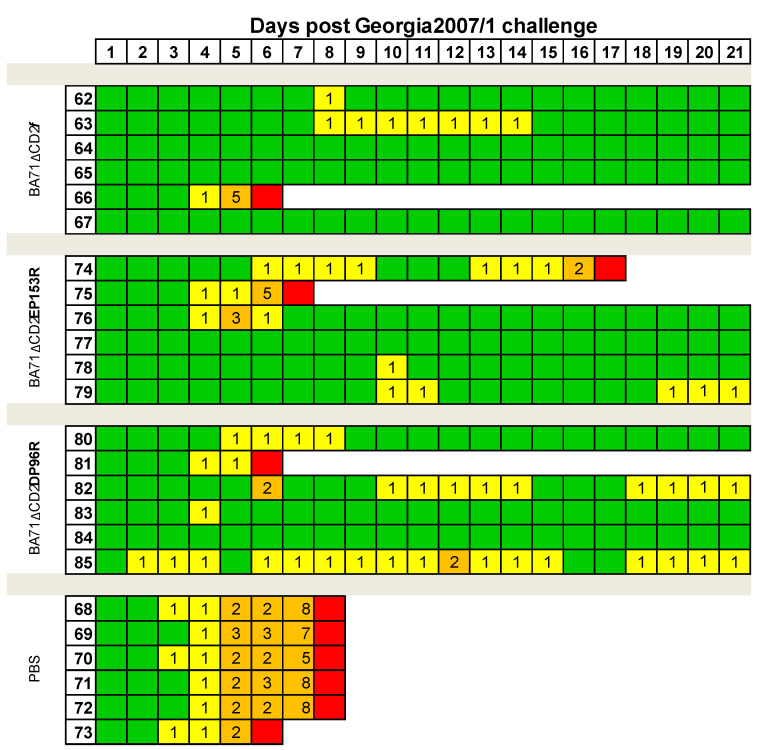
ASF compatible clinical signs observed after challenge. Color palette indicates normal (green), mild (yellow) and high (>41 °C; orange) rectal temperature, while euthanized animals are indicated in red. Clinical scores are indicated with numbers; 1 being low-grade fever (<41 °C) as the only clinical observation.

Pigs that succumbed to Georgia2007/1 challenge (six controls and four immunized with recombinant LAVs) showed clinical signs compatible with acute ASF (Figure 5), including fever, loss of appetite, depression, apathy and a tendency to huddling, as well as erythema and cyanosis. Conversely, surviving pigs did not show severe clinical signs after ASFV challenge (Figure 5). Three out of the six pigs immunized with BA71∆CD2*f* remained clinically normal, while the other two showed only a transient rise in rectal temperature. Thus, pig number 63 showed febricula (~40 °C) from day 8 to 14 pc, while pig number 62 had fever only at day 8 post challenge (rectal temperature < 40.5 °C).

Furthermore, the clinical signs observed after Georgia2007/1 challenge were more evident and prolonged in pigs vaccinated with the double recombinants than with BA71∆CD2*f* (Figure 5), albeit that one out of the six pigs inoculated with BA71∆CD2EP153R (pig number 77) or BA71∆CD2DP96R (pig number 84) never showed any clinical signs. 

**Figure 6:**
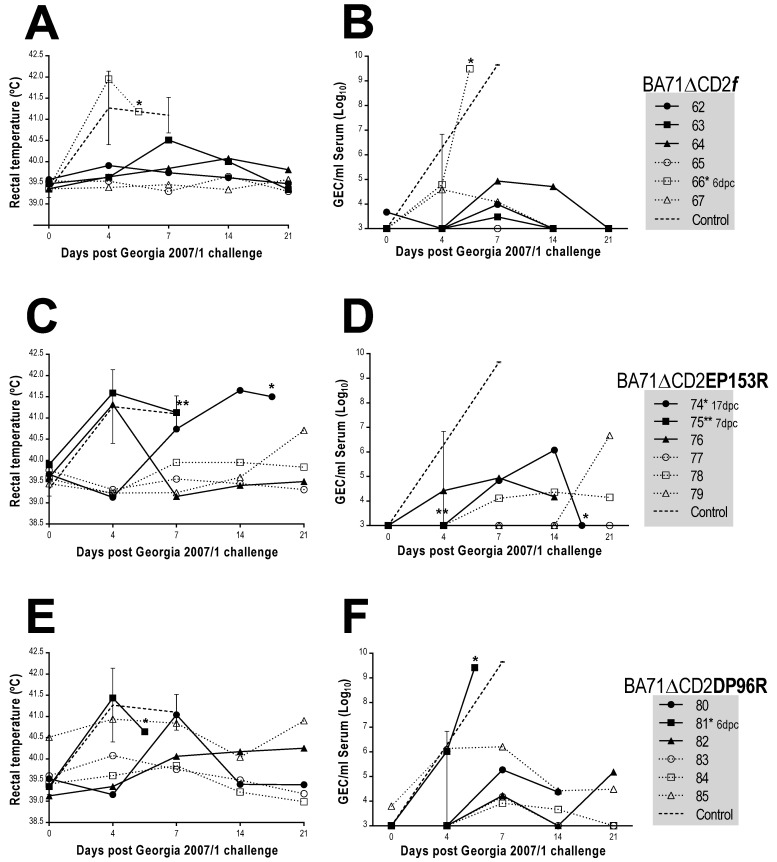
Rectal temperatures measured and ASFV DNA amount found in sera of immunized and control pigs after Georgia2007/1 challenge. Data plotted correspond to individual animals showing rectal temperatures (**A**,**C**,**E**) and virus loads in serum (**B**,**D**,**F**). Upper panels show data corresponding to BA71∆CD2*f* immunized pigs, middle panels to BA71∆CD2EP153R and lower panels to BA71∆CD2DP96R. Average and standard deviation values obtained from control animals are also depicted. Virus titers are plotted on a logarithmic scale as GEC per milliliter of serum, 10^3^ GEC/mL serum being the limit of detection of the assay. * Day of the death of the animals. Last data recorded for pig 75 correspond to day 6pc (death date).

Rectal temperature almost perfectly matched the amount of ASFV DNA detected in serum (Figure 6) after challenge. All control pigs immunized with PBS were euthanized by day 6–8 after the lethal challenge showing significant fever and high amounts of ASFV DNA in sera. As expected, surviving pigs showed significantly lower amounts of ASFV DNA than control pigs (Figure 6). The massive release of immature and defective ASFV particles from lysed infected macrophages at the last stage of the ASFV infection, before the death of the animals, explains the non-linear correlation between GECs (10^10^ GEC/mL) and HAU (~10^8^ HAU/mL) in these samples (not shown). Independently of this, the level of virus in sera of surviving pigs vaccinated with BA71∆CD2*f* (Figure 6A,B) was always 4–5 logarithms below that found in control animals. Furthermore, ASFV presence never lasted more than one week, becoming free of virus by the time of sacrifice, with pig number 65 not showing any detectable ASFV genome in its serum throughout the experiment. The level of virus in sera from pigs immunized with either BA71∆CD2EP153R (Figure 6C,D) or BA71∆CD2DP96R (Figure 6E,F) was also remarkably decreased compared to control animals. Nevertheless, the reduction in virus titers in sera from these animals was slightly lower than that found in the animals immunized with the single mutant BA71∆CD2*f*, and several pigs showed detectable virus by the end of the experiment. 

### 3.3. Evaluation of the Immune Response Induced by BA71∆CD2EP153R and BA71∆CD2DP96R

**Figure 7:**
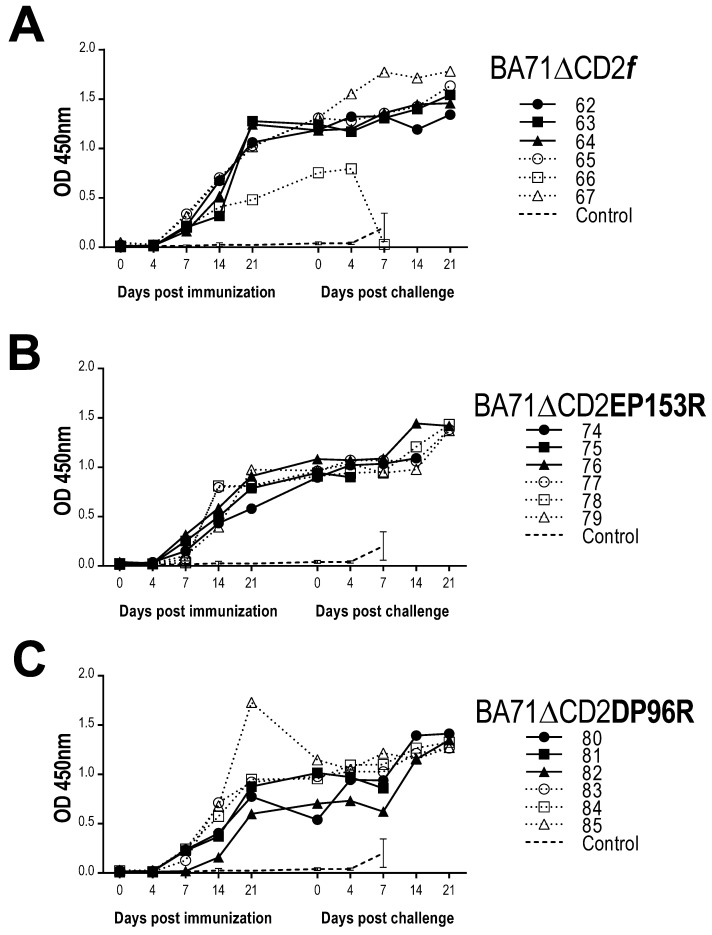
ASFV-specific antibodies detected in serum of pigs immunized with BA71∆CD2*f* (**A**), BA71∆CD2EP153R (**B**) and BA71∆CD2DP96R (**C**), before and after Georgia2007/1 challenge. Individual ELISA optical density (OD) values are shown for each immunization group. Average and standard deviation OD values obtained from the control group are also shown in each figure panel.

**Figure 8:**
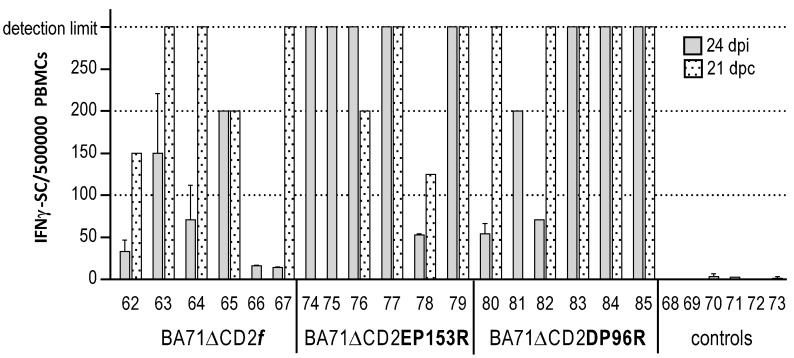
IFNγ-secreting specific T cells found in peripheral blood mononuclear cells (PBMCs) in vitro. Any sample scoring ≥300 spots/500,000 PBMCs received a score of 300, which was considered the limit of our assay resolution.

The antibody kinetics observed before and after challenge were as expected for both controls and BA71∆CD2*f* immunized pigs [12], with the only exception being pig number 66, which showed significant lower ODs than the rest of the animals within the group (Figure 7A). The fact that this pig also showed the lowest ASFV-specific T-cell response (Figure 8) may suggest the possibility of a human/mechanical failure during vaccination, although a simple vaccine failure cannot be ruled out. The severity of the clinical signs observed after challenge (highest score of 12 recorded by day 6, forcing a humane endpoint) might be explained by either an exacerbation effect due to suboptimal immunization as has been described before [2,22] and/or some concomitant health affection. Despite the importance of the ELISA as an indicator of successful immunization, there is not a total correlation between the level of ASFV-specific antibodies measured by ELISA and protection. Thus, pigs 74 and 75 immunized with BA71∆CD2EP153R and pig 81 immunized with BA71∆CD2DP96R died after challenge, despite showing similar antibody levels to surviving pigs at the time of Georgia2007/1 challenge. Conversely, pigs 80 and 82 survived the lethal challenge, despite showing slightly lower ODs. 

The ELISPOT data (Figure 8) confirmed the successful immunization of most of the animals, albeit that again, no correlation was observed between the number of ASFV-specific T cells present in blood and protection (confirming previous results). Thus, pigs 74, 75 and 81 were good responders and died after Georgia2007/1 challenge, while several survivors showed low responses at the time of challenge.

In conclusion, deletion mutants of the attenuated recombinant ASF virus, BA71ΔCD2, decrease vaccine efficacy and do not increase its safety. The fine equilibrium existing between live attenuated ASFV vaccine replication, safety and efficacy requires a more profound study and might provide key lessons for the future.

## Data Availability

The data that support the findings of this study are available from the corresponding author upon reasonable request.
